# Influence of resolution on elevation and slope at watershed scale in Loess Plateau

**DOI:** 10.1186/2193-1801-2-S1-S13

**Published:** 2013-12-11

**Authors:** Wang Chunmei, Yang Qinke, Liu Hongyan, Guo Weiling, David LB Jupp, Li Rui

**Affiliations:** College of Urban and Environmental Science, Northwest University, Xi’an, Shaanxi 710027 China; College of Resources and Environment, Northwestern A&F University, Yangling, Shaanxi 712100 China; Institute of Soil and Water Conservation, Chinese Academy of Sciences And Ministry of Water Resources, Yangling, Shaanxi 712100 China; CSIRO Division of Marine and Atmospheric Research, GPO Box 3023, Canberra, ACT 2601 Australia

**Keywords:** DEM, Slope, Resolution, semi-variogram, "Independent Structures" Model

## Abstract

This article studied the effects of resolution on elevation and slope using Statistical and Geostatistical methods. Xian' Nan watershed in Loess Plateau was taking as the study area. The base data was a 1:10000 topographic map and the resolutions studied in this paper included 5 m, 25 m and 50 m. The results showed that: (1) for elevation and slope data, the mean value, STD value, histogram and semi-variogram changed with resolution reduction. The mean value, STD value became smaller in both elevation and slope cases. Histograms moved to the left which shows there was a decrease of elevation and slope with resolution. The sill for semi-variograms of elevation and slope decreased with resolution reduction; (2) the changes of Mean value; STD and histogram were greater in elevation data than in slope data. (3) By using the Independent Structure model, the semi-variogram could be modeled by 4 components for elevation data and the semi-variogram could be modeled by 3 components for slope data. There was more information in slope than in elevation in the components with short range (short wave-length) information. (4) The influence of resolution reduction was greater in the components with short range, so the degree of influence of resolution reduction was related to the amount of short wave-lengths information. The results of this paper had shown which information was lost with resolution reduction and the reason for the different changes on mean value, STD and histogram for elevation and slope. It could also be used to explain different scaling effects in different terrain areas in the future.

## Introduction

Digital Elevation Models (DEM) is widely used as a digital representation of terrain. Terrain models include important factors for soil erosion and hydrology, etc. [1–3]. DEM resolution is one of the most important factors that influence the ability of a DEM to represent terrain. That is because as resolution becomes coarser, many of the terrain indexes that derived from the DEM will change. Elevation and Slope are two of the most important terrain factors in many study fields [4, 5]. Many researchers have paid attention to the change of terrain factors with DEM resolution reduction such as Chang and Tsai (1991) [6], Gao (1997) [7], Zhang (1999) [8], Wolock (2000) [9] and Wu et al. (2008) [10]. Their results showed that slope tended to become smaller in most of areas with resolution reduction.

But there is not yet sufficient study on why there are differences between terrain factors derived from fine resolution DEM and from coarse resolution DEM and which part of the information has been lost with resolution reduction. Geostatistics has been widely applied to study fields such as vegetation investigation, soil characteristic analyses, etc. Some researchers have used geostatistical analyses to study spatial patterns in topography [11]. In this research the authors studied the structure of terrain and the change in terrain structure with resolution reduction by investigating changes in each component of the semi-variogram. The study uses Xian' Nan watershed as the study area. This watershed is located in Loess Hilly area in Loess Plateau in China. The authors studied the differences between elevation and slope derived from DEMs with resolutions of 5 m, 25 m and 50 m.

The aim of this research is to show which information "disappears" or "reduces" when resolution becomes coarser. This may help explain why terrain factors change with resolution and understand the terrain characteristics which change with resolution.

## Material and methods

### Study area

**Figure 1:**
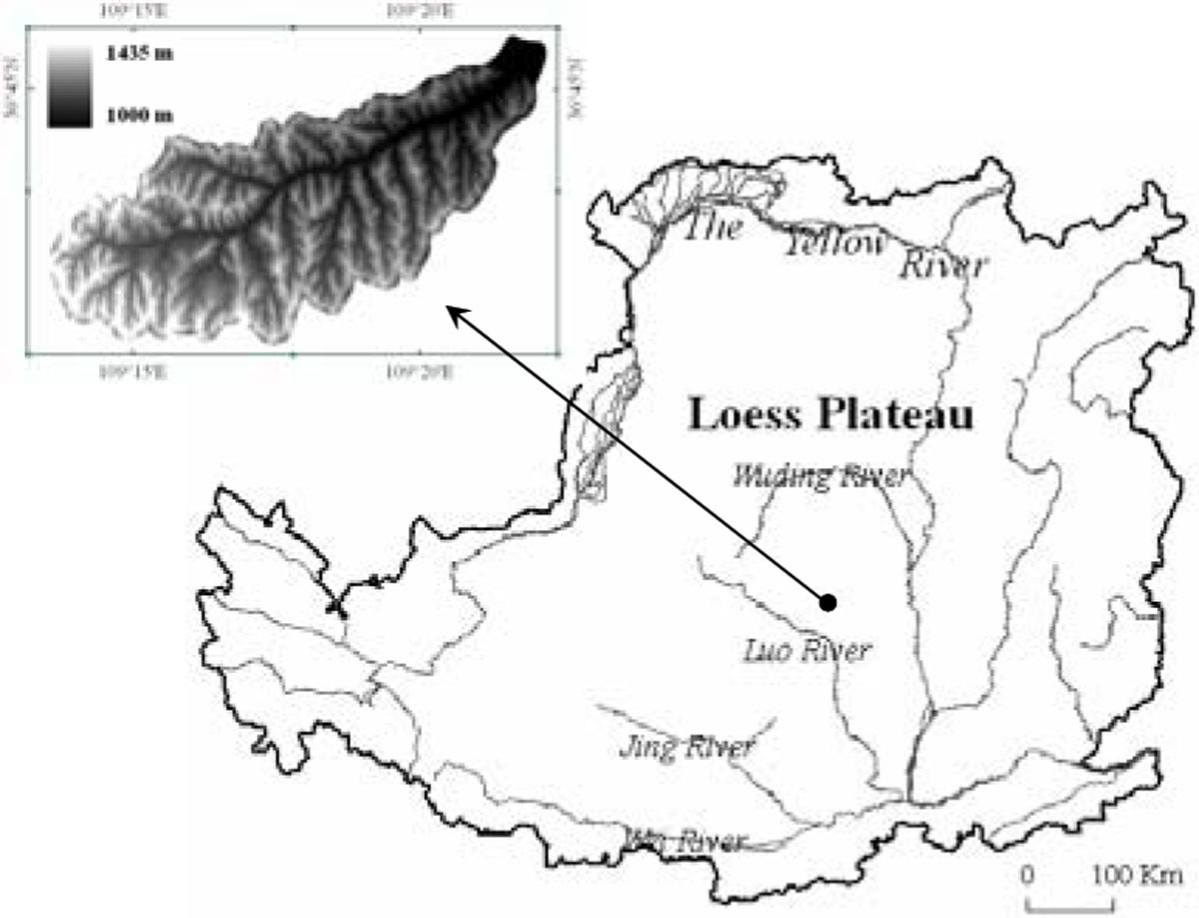
**Location of study area**

The study area is the Xian' Nan watershed located in the Hilly area of the Loess Plateau (E109°11′-109°22′, N36°42′-36°47′) (Figure 1). This watershed covers an area of 44 km^2^ with an average altitude of 1220 m. It is a typical hilly landform in the Loess Plateau with a complex surface configuration and crossing gullies. The ground slope is steep with an average gradient of 28°.

### Base data and data processing

The base data is a topographic map at 1:10,000 scale issued by China's National Bureau of Surveying and Mapping in 1981 with a contour interval of 5 m which covers the whole study area. The data processing includes topographic map digitizing, projection transformation, check in elevation values, check in river directions and Lake Boundaries. The projection for the base data is Krasovsky_1940_Albers. Longitude of the Central Meridian is E105°. The first Standard Parallel is N25° and the second Standard Parallel is N47°. Latitude of projection Origin is 0°. False Easting and False Northing are both 0 m. The projection is an equal-area projection. The grid cells are "square" and in meters.

DEMs with resolution of 5 m, 25 m and 50 m were built using ANUDEM which were developed in Australia by Hutchinson [12]. ANUDEM interpolates the data from the topographic maps to finer resolutions in down scaling steps from coarser resolutions. The ANUDEM software interpolates the DEM where there is no source data using minimum curvature in order to make the surface as smooth as possible. The finest resolution used here is 5 m because 5 m is found to be the most suitable finest resolution according to the ANUDEM rules for topographic maps with scale of 1:10,000. The coarser DEMs seem to be similar with those obtained by up scaling the finer DEMs using low pass filtering to some extent, however it is not fully the same. Research is under way into the nature of the differences. The parameter values used in ANUDEM are showed in Table 1.

**Table 1:** Parameters of ANUDEM

Maximum iterations	Profile curvature	Cell size (m)
40	0.7	5, 25, 50

### Information statistics for elevation and slope

#### Slope calculation

In this research slope is calculated using the following definition:

Where  refers to slope; p and q refer to gradient field components. In a digital image, p and q are both calculated using the high pass filters as following.

Where "h" is the resolution step or grid cell size for the data.

### Histogram intersection

Swain and Ballard [13] efficiently recognized objects by matching their color histograms using the histogram intersection (HI) method. In this research the author used Histogram Intersection (HI) to evaluate histogram similarity.

HI is calculated by:

Where HI(X, Y) is Histogram Intersection of two histograms X and Y;  and  are frequency values of X and Y at slope value of i. The values for HI(X, Y) range from 0% to 100%. Histograms are more similar to each other if HI(X, Y) is larger. If two histograms are totally the same, HI(X, Y) equals to 100%.

#### Reduction rate of mean value and STD

Mean value Reduction Rate and STD reduction rate for both elevation and slope are calculated here to refer to absolute value of mean value and STD change per reduction of resolution in meter. It will be used to help quantitatively analyze mean value and STD change with resolution. It is calculated by:

Where  is Mean value Reduction Rate;  is mean value with coarser resolution of ;  is mean value with finer resolution of ;  is STD Reduction Rate;  is STD value with coarser resolution of ;  is STD value with finer resolution of .

### Geostatistical analysis

#### Covariance and semi-variogram

In geostatistics, the mathematical expression for covariance and the semi-variance function is:

 is the covariance function and  is the semi-variance function; (x, y) stands for the spatial coordinators of the tested point of the slope; Z(x, y) stands for the data of the tested point;  and  stands for the interval in x and y direction between two tested points (in 1D cases ), m is the mean value over the image. We will assume that the covariance is stationary and the mean (m) is a constant over the image.

In this case the relationship between the two expressions is:

 is the variance of the image data which needs to be spatially stationary [14].

#### Modeling covariance and semi-variogram

In this article a form of model for the semi-variogram called the "Independent Structures" model [14] (sometimes it is called "Nested Model") is assumed. In this model the original field  is considered to be composed of N "independent" fields, the covariance between the N fields is zero.

So the total variance of Z is:

In the "Independent Structures" Model, the covariance function and semi-variogram fuction for Z is given by:

 refers to the ranges of the component semi-variograms and:

In this way, the first component is the one with greatest "roughness" or high spatial frequency content effect and the last is the one with greatest low frequency (regional) effect. This representation models the data with different "scale" components with  representing scale components with decreasing map scale.

In this paper the authors assume that the base semi-variogram model is the radial 2D Gaussian, the covariance for each component is:

The radial distance is ;  is the variance of the component j; The quantity  is taken as the range of component j and the component has the form of a correlation function.

#### Effect of filtering on the covariance and semi-variogram

Assuming that the function Z that is being filtered has mean zero and finite variance, the covariance function for Z is:

If Z is filtered by a filter , then:

Where  the covariance of the filtered functions and  is the semivariogram of the filtered function.

If the base semi-variogram model is radial 2D Gaussian, and the smoothing kernel is a normalized radially symmetric 2D Gaussian with range S, then:

FWHM (Full Width Half Maximum) is the width of the filter at half height. H is the height of the filter.

In this case the covariance of filtered function is:

The covariance of the filtered function is the same type as before however it has reduced variance and increased range.

#### Model efficiency analysjs

In order to evaluate the result of the Independent Structures model of the semi-variogram, the model efficiency coefficient (ME) which was proposed by Nash and Sutcliffe [15] was used in this research.

ME is calculated by:

Where ME is the model efficiency,  is the observed value,  is the predicted value,  is the mean observed value. Values for ME range from -∞ to 1. The closer ME is to 1, the better the model will predict individual values.

## Results and analysis

### Mean elevation and slope change with resolution

Table 2 shows the mean value and Standard deviation (STD) of both elevation and slope. Elevation in Xian'nan Watershed does not change greatly with resolution reduction but slope seems to be largely influenced by resolution. Mean elevation and STD of elevation tend to decrease slightly with resolution reduction with a reduction rate of 0.03 m/m. Mean slope and STD of slope tend to decrease with resolution reduction more intensively than elevation and the reduction rates are different with different resolution changes. The mean slope reduction rate with resolution from 5 m to 25 m of 0.3°/m is the largest (Table 3). This means that when resolution becomes 1 meter coarser, the mean slope will decrease by 0.3° which is large.

**Table 2:** Mean and Std of elevation and slope

	Mean value		STD	
**Resolution (m)**	**Elevation (m)**	**Slope (°)**	**Elevation (m)**	**Slope (°)**
5	1220.1	28.5	74.0	11.1
25	1219.5	22.7	73.4	8.8
50	1218.9	18.5	72.9	7.4

**Table 3:** Mean value and STD reduction rate

	Mean value		STD	
	**Elevation (m)**	**Slope (°)**	**Elevation (m)**	**Slope (°)**
5 m-25 m	-0.03	-0.29	-0.03	-0.12
25 m-50 m	-0.03	-0.17	-0.03	-0.07

### Histograms of elevation and slope changes with resolution

**Figure 2:**
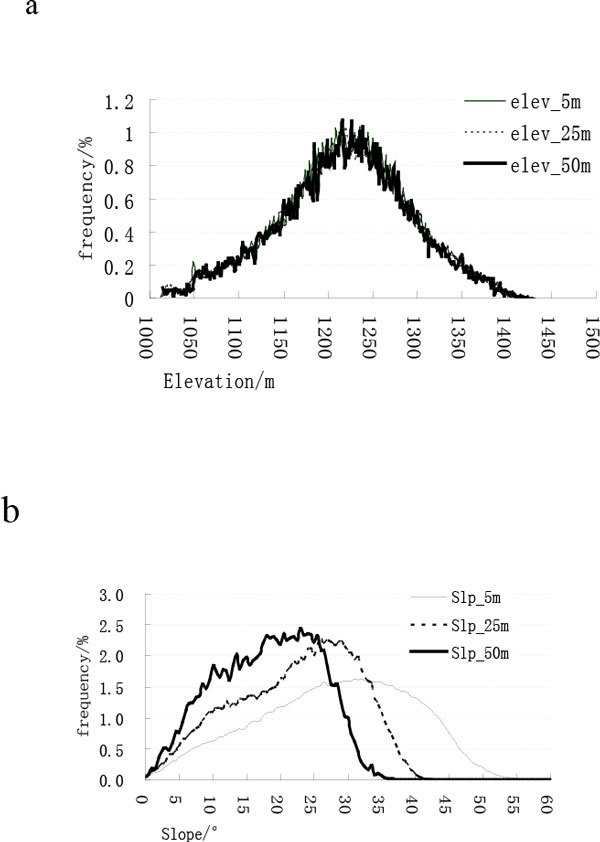
**a Histograms of elevation with varied resolution**. b Histograms of slope with varied resolution

Histograms of elevation with varied resolutions of 5 m, 25 m and 50 m are shown in Figure 2a. Histograms for elevation mix together because they are quite similar to each other. Elevation histogram distribution changes little because of the resolution reduction. HI in Table 4 clearly shows that the HI values between elevation histograms with 25 m, 50 m resolution and elevation histogram with 5 m resolution are both larger than 90%.

**Table 4:** HI comparing with 5 m data (%)

	5 m	25 m	50 m
DEM	100	96.258	94.0111
Slope	100	73.774	55.7426

There seems to be a great difference between slope histograms with 25 m, 50 m resolution and the slope histogram with 5 m resolution (Figure 2b). As resolution reduces, the histograms of slope move to the left side which is lower. HI values between the slope histograms with 25 m and 50 m resolution and the slope histogram with 5 m resolution are 73.8% and 55.7% which shows the great influence of resolution on the histogram of slope.

### Changes in semi-variogram of elevation and slope with resolution

#### Independent structures of semi-variogram

**Figure 3:**
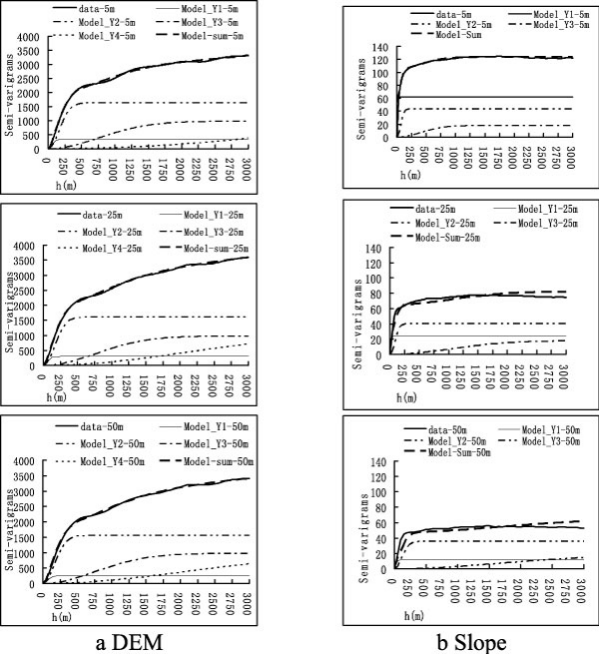
**Independent model of semi-variogram**

Semi-variogram of elevation with resolution of 5 m, 25 m and 50 m are calculated and modeled using the Gaussian model. These are plotted in Figure 3a. The elevation semi-variogram in the Xian'nan watershed has been modeled with 4 components which are labeled as Y1, Y2, Y3, and Y4. Sill and range for each component are shown in Table 5. The ranges are ordered by Y1<Y2<Y3<Y4 as in the independent model. Y4 indicates information that of longer wave-length. Y4 seems to be a trend component and has not reached a sill inside the maximum lag of semi-variance. This component is "unstable" and cannot be treated in the same way as the other components. The sills are ordered as: Y1<Y3<Y2. Y1 indicates information relating to short wave-length. In the elevation data in the study area, the short wave-length information accounts for much less variance than the long wave-length information.

**Table 5:** Sill and range of elevation semi-variogram

	Resolution (m)	Range	Sill
	5	140.24	328.23
Y1	25	148.64	292.19
	50	164.54	238.44
	5	404.38	1629.60
Y2	25	407.37	1605.78
	50	413.44	1558.99
	5	1866.01	977.49
Y3	25	1866.66	976.81
	50	1867.99	975.42
	5	50315.37	35390.90
Y4	25	5212.52	1133.83
	50	5058.91	977.95
	5	2410.63	2935.33
Model-Sum	25	2422.67	2874.78
	50	2445.98	2772.84

*Model-sum is the sum of range and sill of each component except Y4 because Y4 has not Come to a range inside maximum lag of semi-variance.

Semi-variograms of slope with resolution of 5 m, 25 m and 50 m were calculated and modeled. These are plotted in Figure 3b. The slope semi-variogram in Xian'nan watershed has been modeled using 3 components which are labeled as Y1, Y2, and Y3. Sill and range of each component are shown in Table 6. The range is ordered by Y1<Y2<Y3 in the independent model. All of the three components have reached a sill inside the maximum lag of semi-variance. The sill values are ordered as: Y3<Y2<Y1 with 5 meter resolution. In slope data in the study area, the short wave-length information accounts for more variance than the long wave-length information.

**Table 6:** Sill and range of slope semi-variogram

	Resolution (m)	Range	Sill
	5	32.88	61.71
Y1	25	39.58	23.90
	50	49.68	11.18
	5	150.19	43.51
Y2	25	207.23	40.44
	50	284.18	35.76
	5	997.53	17.97
Y3	25	2503.71	17.94
	50	4006.32	17.88
	5	1180.60	123.18
Model-Sum	25	2750.52	82.27
	50	4340.18	64.83

#### The effect of resolution on the independent structures of semi-variogram

The effect of resolution reduction is quite similar to low pass filter. If we treat the resolution reduction as a Gaussian low pass filter, the semi-variograms with coarser resolution can be predicted from the fine resolution using the method discussed in this paper. The sill and range in each component can be predicted. In this paper, the authors predicted the coarser resolution variograms of each component from the 5 m resolution variograms. The ME of the model is shown in Table 7. The ME values are all above 0.65.

**Table 7:** ME of independent structure model

Resolution	Elevation	Slope
5	0.9997	0.9920
25	0.9994	0.8231
50	0.9987	0.6743

The influence of resolution reduction is smallest on long wave-length components and is largest for Y1 in both elevation and slope cases. Table 5 and Figure 3a show that in the elevation case, the sill value of Y1 reduced to 72.6% of the 5 m case when resolution is 50 m. Y2 and Y3 seem to have be stable and the reduction of sill when the resolution is 50 m is less than 5% of the 5 m case. Table VI and Figure 3b show that in slope case, the sill value of Y1 reduced to 18.1% of the 5 m case when resolution is 50 m. The sill value of Y2 reduced to 82.2% of the 5 m case when the resolution is 50 m and the sill value of Y3 reduced to99.5% of the 5 m case when the resolution is 50 m.

Comparing the elevation and slope cases, the influence of resolution reduction on slope is much larger than on elevation especially in the Y1 components. The Sill (Model sum) of elevation reduced only to 94.4% of the 5 m case but the sill (Model sum) of slope reduced to 52.6% of the 5 m case. The reason is that the process of slope calculation acts as a high pass filter. More short wave-length information is left in the image after the slope is calculated. Therefore there is much more short wave-length information in slope data than in elevation data. The low pass filter and the resolution reduction effect greatly influence the short wave-length information.

## Discussion

ME of slope is lower than ME of elevation. It may because that the Gaussian Model is not the most appropriate one for slope. In this paper the main purpose is to show the scale effect which has been done. Work on appropriate model for slope is investigated by the authors.

In this paper the authors modeled the effects of resolution reduction on the semi-variogram using a Gaussian filter. Although the way ANUDEM changes with scale do not behave exactly the same way as a Gaussian filter, the model can work quite well. In the future, more effort is being put into the study of the differences between the two.

The semi-variograms in this paper has been modeled using Independent structure model. The model allows us to clarify the way resolution influence DEM data. In further study this would be applied to different terrain types.

In further study, the semi-variogram of each component will be mapped out using kriging to show the nature of different information in each component.

## Conclusions

In this paper the independent structure model was used to model the semi-variograms of elevation and slope data with resolution of 5 m, 25 m and 50 m. The results showed how the short wave-length information disappeared or weakened as resolution reduced. These results can explain which component of the information has changed with resolution reduction. By calculating the mean value, STD and histogram of both elevation and slope at different resolutions, it is clear that the influence of resolution on elevation is less than on slope. The reason is that the short wave-length information accounts for more variance in slope data than in elevation data. Resolution reduction or low pass filtering influence the short wave-length information the most.
